# Endothelial Mesenchymal Transition in Hypoxic Microvascular Endothelial Cells and Paracrine Induction of Cardiomyocyte Apoptosis Are Mediated via TGFβ_1_/SMAD Signaling

**DOI:** 10.3390/ijms18112290

**Published:** 2017-10-31

**Authors:** Isabella Sniegon, Mona Prieß, Jacqueline Heger, Rainer Schulz, Gerhild Euler

**Affiliations:** Institute of Physiology, Justus Liebig University, 35392 Giessen, Germany; isabella.sniegon@gmx.de (I.S.); mona.priess@physiologie.med.uni-giessen.de (M.P.); jacqueline.heger@physiologie.med.uni-giessen.de (J.H.); rainer.schulz@physiologie.med.uni-giessen.de (R.S.)

**Keywords:** fibrosis, TGFβ, ischemia, cardiac remodeling, endothelial cells, cardiomyocytes, apoptosis

## Abstract

Cardiac remodeling plays a crucial role in the development of heart failure after mycocardial infarction. Besides cardiomyocytes, endothelial cells are recognized to contribute to cardiac remodeling. We now investigated processes of endothelial mesenchymal transition (EndoMT) in microvascular endothelial cells of rat (MVEC) under hypoxia and paracrine effects on ventricular cardiomyocytes of adult rat. Exposure of MVECs to hypoxia/reoxygenation enhanced TGFβ/SMAD signaling, since phosphorylation, and thus activation, of SMAD1/5 and SMAD2 increased. This increase was blocked by inhibitors of TGFβ receptor types ALK1 or ALK5. Exposure of ventricular cardiomyocytes to conditioned medium from hypoxic/reoxygenated MVECs enhanced SMAD2 phosphorylation and provoked apoptosis in cardiomyoyctes. Both were blocked by ALK5 inhibition. To analyze autocrine effects of hypoxic TGFβ signaling we investigated EndoMT in MVECs. After 3 days of hypoxia the mesenchymal marker protein α-smooth muscle actin (α-SMA), and the number of α-SMA- and fibroblast specific protein 1 (FSP1)-positive cells increased in MVECs cultures. This was blocked by ALK5 inhibition. Similarly, TGFβ_1_ provoked enhanced expression of α-SMA and FSP1 in MVECs. In conclusion, hypoxia provokes EndoMT in MVECs via TGFβ_1_/SMAD2 signaling. Furthermore, release of TGFβ_1_ from MVECs acts in a paracrine loop on cardiomyocytes and provokes apoptotic death. Thus, in myocardial infarction hypoxic endothelial cells may contribute to cardiac remodeling and heart failure progression by promotion of cardiac fibrosis and cardiomyocytes death.

## 1. Introduction

Myocardial infarction provokes cardiomyocyte death and scar formation in the infarct area, but also causes diverse remodeling processes, like cardiomyocytes hypertrophy or fibrosis. Cardiac fibrosis is not only established by resident fibroblasts, but also by transition of other cell types into fibroblasts. In this context, a pivotal role is attributed to the transition of endothelial to mesenchymal cells (EndoMT). The group of Zeisberg et al. [1] has shown that the proportion of endothelial derived fibroblasts increases to around 25% after pressure overload, and hypoxic conditions promote EndoMT in human coronary endothelial cells [2]. Furthermore, hypoxic conditions can disturb the barrier function of the endothelium, since cell–cell contacts are reduced in hypoxic rat coronary endothelial cells due to disarrangement of the actin cytoskeleton and the loss of cadherins at the cell surface. This phenomenon leads to reductions of adherence junctions [3]. Similar to this situation, also during EndoMT endothelial cells lose the expression of characteristic surface endothelial markers, such as platelet endothelial cell adhesion molecule (PECAM-1/CD31) or vascular endothelial (VE)-cadherin, and consequently the organization of compact cell layers is disrupted. Therefore, reduced barrier function under hypoxic conditions may also be a result of EndoMT [4], and may thus enable enhanced crosstalk of the endothelium and cardiomyocytes. Thereby, the endothelial cells can exercise substantial control over the contractility and growth of cardiomyocytes [5,6].

Members of the TGFβ family are the best known inducers of EndoMT [4]. They can signal through type I and type II serine/threonine kinase receptors, and thereby activate the canonical SMAD pathway. In endothelial cells, TGFβ activates two distinct type I receptors, also called activin receptor like kinase, ALK5 and ALK1, either resulting in phosphorylation and activation of the transcription factors SMAD2 and 3, or SMAD 1 and 5, which then binds to their specific target gene promoters [7]. Both receptor types can convey EndoMT, since genetic ablations of either ALK 1 or 5 abolish EndoMT in mice [4].

After myocardial infarction levels of cardiac TGFβ are enhanced. In this context, reductions of fibrosis after myocardial infarction have been shown in SMAD3 knock out mice, thereby demonstrating involvement of TGFβ/SMAD-signaling in ischemia/reperfusion-induced cardiac fibrosis [8]. But TGFβ_1_ can also modulate cardiomyocyte hypertrophy. Thus, TGFβ_1_ promotes cardiac remodeling via its influence on cardiac hypertrophy and fibrosis [9,10]. In the heart TGF can be produced by cardiomyocytes themselves or by endothelial cells. Under hypoxic conditions endothelial cells release bio-active TGFβ [11], and just recently Xu et al. [2] demonstrated in human coronary artery endothelial cells autocrine induction of EndoMT via TGFβ_2_/SMAD signaling under hypoxic conditions. Thus, the release of TGFβ from endothelial cells may either promote EndoMT in an autocrine loop, but it may also have paracrine effects on cardiomyocytes. 

Therefore, in this study we analyzed effects of hypoxia on TGFβ signaling and EndoMT in microvascular endothelial cells of rat as well as paracrine effects on cardiomyocytes. 

## 2. Results

### 2.1. Establishment of Pure Microvascular Endothelial Cell Cultures (MVECs)

**Figure 1:**
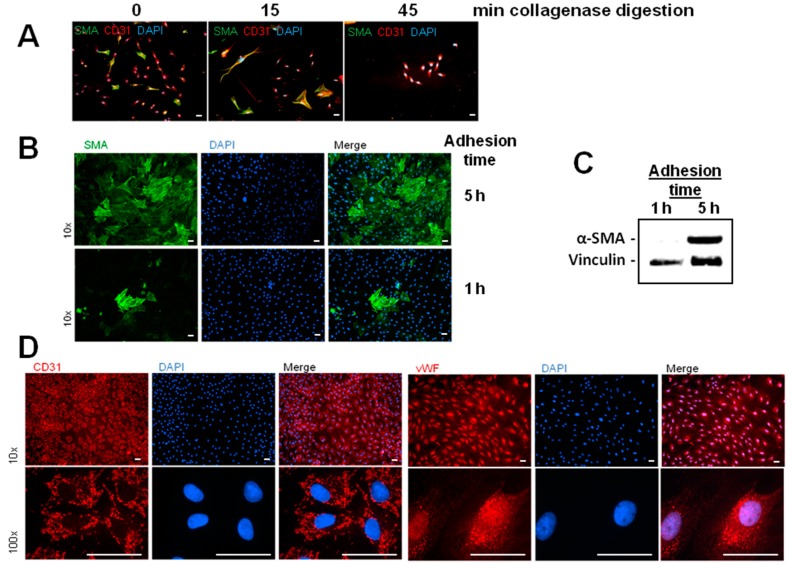
Establishment of pure endothelial cell cultures. (**A**) Endothelial cells were isolated as described in the method section. After separation of cardiomyocytes from other cardiac cells, the remaining cells were redigested with collagenase (400 mg/L) for 0, 15, or 45 min. Then cells were plated on culture dishes and immnuofluorescene was performed using antibodies against α-SMA (green) and CD31 (red), as well as DAPI was used for nuclear staining (blue); (**B**–**D**) cells with a redigestion time of 45 min were plated on culture dishes. After one or five hours adhesion time, cells were rigorously washed, cultured to 95% confluence and once passaged. Either immunofluorescence or Western blots were performed when the cell layer reached around 95% confluence; (**B**) immnuofluorescene was performed using antibodies against α-SMA (green). Nuclei were staind with DAPI (blue); (**C**) Western blot of total protein using α-SMA antibodies and vinculin antibodies for loading control; (**D**) immnuofluorescene was performed using antibodies against CD31 (red) or von Willebrandt factor (vWF, red) to detect endothelial cells. Nuclei were stained with DAPI (blue). Scale bars: 50 µm

MVECs were isolated from rat hearts by collagenase digestion and differential centrifugation as described in the method section. After the last centrifugation step an MVEC enriched fraction was obtained. A critical point for the purity of this MVEC enriched cell fraction was the time of collagenase digestion (Supplementary Figure S1). Using an α-smooth muscle actin (α-SMA) antibody as mesenchymal marker, CD31 antibody as endothelial marker, and DAPI for nuclear staining in immunofluorescence histology, we could demonstrate enrichment of MVECs by increasing collagenase redigestion to 45 min (Figure 1A). Another critical point for purification of endothelial cells was the adhesion time after plating the cell fraction on culture dishes. After adhesion, cultures were washed thoroughly and cultured to 95% confluence. Cells were then passaged once, recultured again to 95% confluence before cell culture purity was determined (Supplementary Figure S1). As can be seen in Figure 1C, α-SMA could be detected in Western blots of cultures with adhesion times of 5 h, but not in cultures with 1 h of adhesion time. Also, in immunohistology α-SMA positive cells were tremendously decreased in cell cultures with 1 h adhesion time, reaching a level of 8.9 ± 2.2% of total cell number (*n* = 8) (Figure 1B). This indicates that MVECs adhere faster (within 1 h) to the culture dish, so that α-SMA expressing, mesenchymal cells can be washed off. The remaining cells with greater than 90% purity could be identified as endothelial cells, since they express the typical marker CD31 and von Willebrandt factor (vWF) (Figure 1D).

### 2.2. Induction of TGFβ_1_/SMAD Signaling in Hypoxic Reoxygenated MVECs

**Figure 2:**
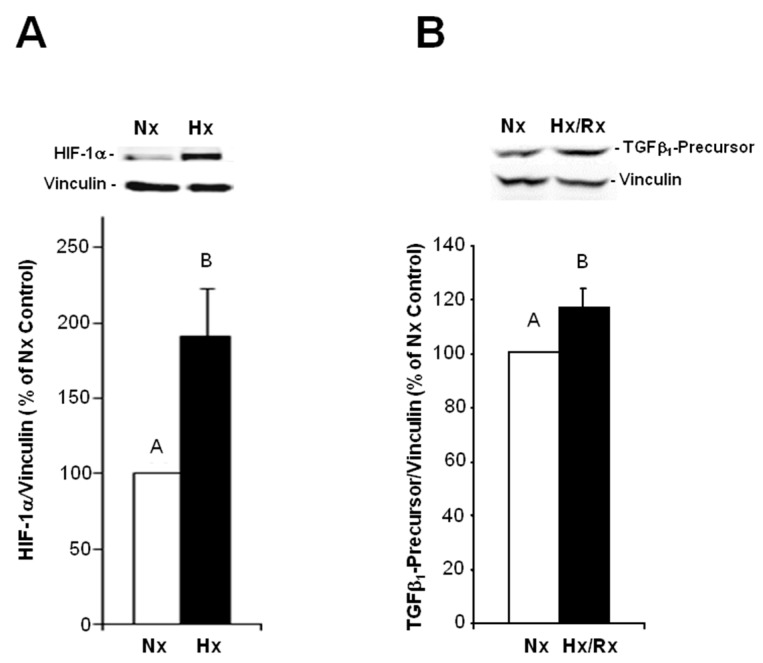
Induction of hypoxia inducible factor 1α (HIF-1α) and TGFβ_1_ by hypoxia. MVECs were cultured under hypoxic conditions (Hx) in an atmosphere containing 95% N_2_/5% CO_2_ and then reoxygenated (Rx). Total proteins were extracted (**A**) after one hour hypoxia for detection of HIF-1α, and (**B**) after one hour hypoxia and 15 min of reoxygenation for detection of TGFβ_1_. Western blots were performed using antibodies against HIF-1α, TGFβ_1_ or vinculin as loading control. Representative Western blots, and quantification of HIF-1α expression are depicted. (**A**) HIF-1α/vinculin ratios are expressed as percent increase relative to normoxic controls, and are presented as means ± SE of *n* = 5–7 independent culture preparations; (**B**) TGFβ_1_/vinculin ratios are expressed as percent increase relative to normoxic controls, and are presented as means ± SE of *n* = 4 independent culture preparations. Uppercase letters indicate significant differences between groups with *p* ≤ 0.05.

MVECs were incubated in a hypoxic chamber for one hour followed by reoxygenation. Different time points of hypoxia/reoxygenation were used to detect the sequential appearance of signal molecules. In suppl. Figure 2 the respective times of hypoxia and reoxygenation are depicted. Hypoxic conditions were achieved by incubation of cells in an atmosphere containing 95% N_2_/5% CO_2_. Under this condition, oxygen deprivation reached a level of <1% O_2_, measured with an oxygen sensor. To verify hypoxic conditions on a cellular level, HIF-1α expression was determined in Western blots. As can be seen in Figure 2A HIF-1α was significantly increased after one hour of hypoxia to 191.6 ± 31.6% vs. normoxic controls (*p* ≤ 0.05, *n* = 5–7). 

**Figure 3:**
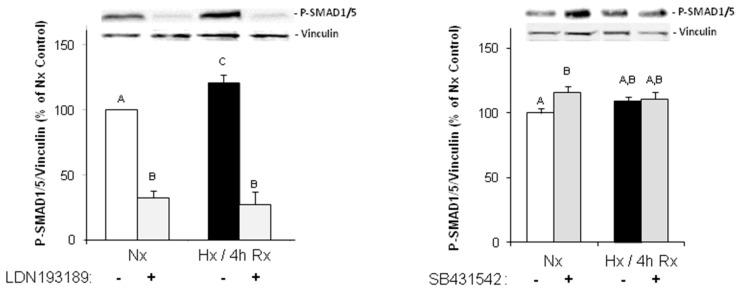

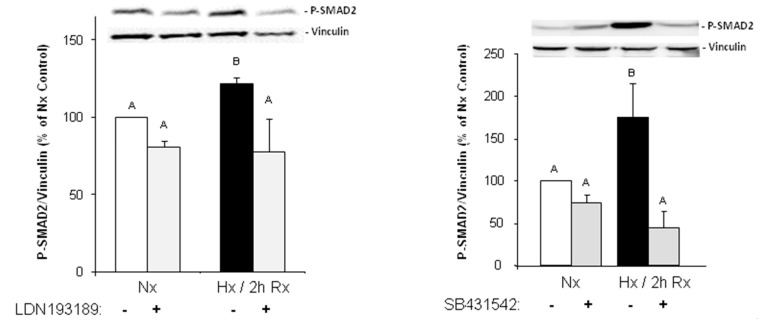
Activation of SMAD-signaling in hypoxic reoxygenated endothelial cells. Endothelial cells were exposed to one hour hypoxia or normoxia (Nx), followed by two or four hours of reoxygenation (Rx) in presence or absence of the ALK1 inhibitor LDN193189 (1 μg/mL) or the ALK5 inhibitor SB431542 (1 μM). Then Western blots were performed using antibodies against phospho-SMAD1/5, phospho-SMAD2, or vinculin as loading control. Representative Western blots, and quantification of protein expression are depicted. For quantification, phospho-SMAD1/5/vinculin or phospho-SMAD2/vinculin ratios are expressed as percent increase relative to normoxic controls, and are means ± SE of *n* = 13 or *n* = 3 (for P-SMAD2 ± SB431542) independent culture preparations. Uppercase letters indicate significant differences between groups with *p* ≤ 0.05.

Under these hypoxic conditions enhancement of TGFβ_1_ expression could be detected in western blots: After one hour hypoxia and 15 min of reoxygenation TGFβ_1_-precursor protein expression increased to 122.2 ± 8.4% (*p* ≤ 0.05 vs. normoxic control, *n* = 4) (Figure 2B). As a functional consequence of TGFβ_1_ release, activation of the SMAD pathway was observed: After 1 h hypoxia and 4 h of reoxygenation, SMAD1/5 was phosphorylated to 120.7 ± 6.4% (*p* ≤ 0.05 vs. normoxic control, *n* = 13) (Figure 3). This activation/phosphorylation of SMAD1/5 was abolished when cells were preincubated with the ALK1-receptor inhibitor LDN193189 (1 μg/mL) whereas the ALK5-receptor inhibitor had not influence on SMAD1/5 phosphorylation. An increase in SMAD2 phosphorylation could be detected within 1 h hypoxia and 2 h of reoxygenation to 175.9 ± 39.2% (*p* ≤ 0.05 vs. normoxic control, *n* = 3) (Figure 3). This increase could be blocked by ALK1 and ALK5 inhibitors (Figure 3).

### 2.3. Release of TGFβ_1_ from Hypoxic Endothelial Cells Induces Apoptosis in Cardiomyocytes

**Figure 4:**
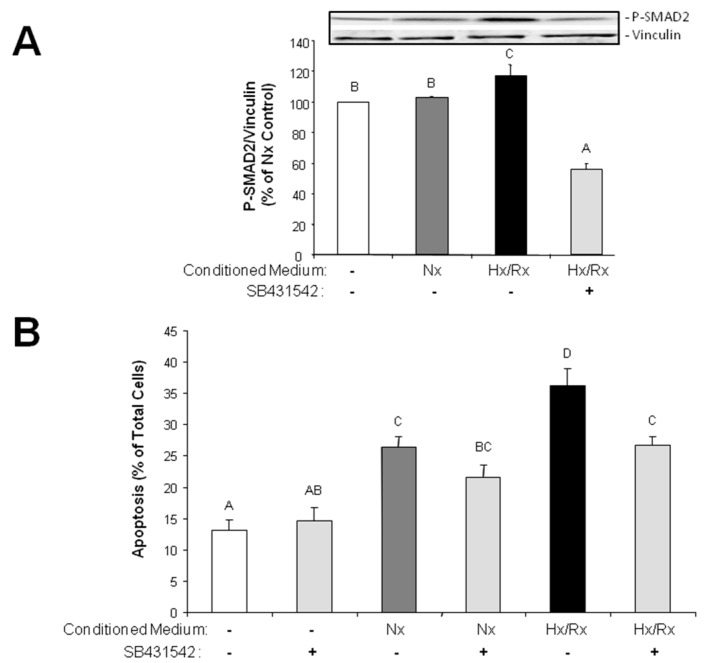
Apoptosis induction in ventricular cardiomyocytes by conditioned medium from hypoxic/reoxygenated endothelial cells via ALK5 receptor. Endothelial cells were exposed to one hour hypoxia or normoxia and two hours reoxygenation. Then conditioned medium of normoxic (Nx) or hypoxia/reoxygenated (Hx/Rx) cells were added to ventricular cardiomyocytes in presence or absence of the ALK5 inhibitor SB431542 (1 μM). Control cardiomyocytes were cultured in normal normoxic medium for cardiomyocytes. (**A**) After two hours total protein was extracted and Western blots performed using phospho-SMAD2 or vinculin antibodies. Representative Western blots, and quantification of protein expression are depicted. For quantification phospho-SMAD2/vinculin ratios are expressed as percent increase relative to normoxic controls, and are means ± SE of *n* = 8 independent culture preparations. Uppercase letters indicate significant differences between groups with *p* ≤ 0.05; (**B**) After 24 h the rate of apoptotic cardiomyocytes was determined by staining nuclei with Hoechst 33258 for detection of chromatin condensation. Number of apoptotic cells is calculated as percent of total cells and are presented as means ± SE of *n* = 4 independent culture preparations. Uppercase letters indicate significant differences between groups with *p* ≤ 0.05.

Since TGFβ_1_ has been shown to provoke apoptosis in ventricular cardiomyocytes of adult rat, we presumed that conditioned medium from hypoxic/reoxygenated endothelial cells may enhance cardiomyocyte cell death. In order to prove this hypothesis we added conditioned culture medium of endothelial cells that underwent one hour of hypoxia and two hours of reoxygenation to isolated ventricular cardiomyocytes of adult rat. First, we analyzed if the classical TGFβ_1_pathway is activated under this condition. Indeed, conditioned medium from hypoxic/reoxygenated endothelial cells enhanced phosphorylation of SMAD2 in ventricular cardiomyocytes within two hours to 117.3 ± 7.2% (*p* ≤ 0.05 vs. control, *n* = 8) (Figure 4A). This induction of SMAD2 phosphorylation was inhibited by the ALK5 inhibitor SB431542 (1 μM). 24 h after incubation of cardiomyocytes with conditioned medium from hypoxic/reoxygenated endothelial cells we determined apoptosis by detection of chromatin condensation in the nuclei. Conditioned medium from hypoxic/reoxygenated endothelial cells increased the number of apoptotic cardiomyocytes from 13.1 ± 1.2% in controls to 36.3 ± 2.6% (*p* ≤ 0.05 vs. control, *n* = 4) (Figure 4B). However, conditioned medium from normoxic cells also enhanced apoptosis in cardiomyocytes, although not to the same extent as the medium from hypoxic/reoxygenated endothelial cells (26.4 ± 1.7%, *p* ≤ 0.05 vs. control, *n* = 8). Addition of the ALK5 inhibitor to conditioned medium from hypoxic/reoxygenated cells reduced the number of apoptotic cardiomyocytes to 26.8 ± 1.4% (*p* ≤ 0.05 vs. cells in conditioned medium from hypoxic/reoxygenated cells, *n* = 4). This was the same level as was found induced by normoxic conditioned medium (*p* ≤ 0.05 vs. cells in conditioned medium from hypoxic/reoxygenated cells, *n* = 4) (Figure 4B). 

### 2.4. EndoMT under Hypoxic Conditions

**Figure 5:**
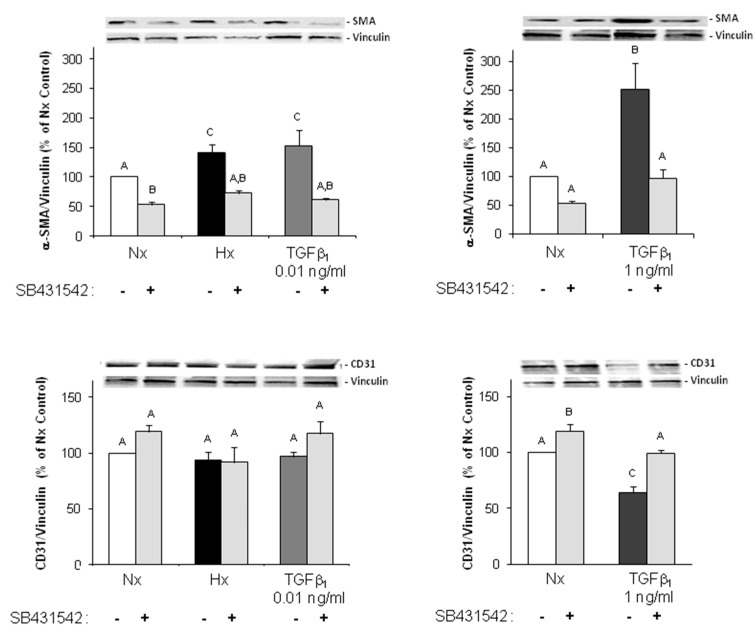
Hypoxia and TGFβ_1_ enhance α-SMA expression via SMAD2-signaling in MVECs. Endothelial cells were exposed to three days normoxia (Nx), hypoxia (Hx) or TGFβ_1_ in presence or absence of the ALK5 inhibitor SB431542 (1 μM). Then Western blots were performed using antibodies against α-SMA, CD31, and vinculin as loading control. Representative Western blots, and quantification of protein expression are depicted. For quantification, α-SMA/vinculin or CD31/vinculin ratios are expressed as percent increase relative to untreated normoxic controls, and are means ± SE of *n* = 8 independent culture preparations. Uppercase letters indicate significant differences between groups with *p* ≤ 0.05.

To determine if hypoxia induces EndoMT we analyzed the expression of the endothelial marker CD31 and the mesenchymal marker α-SMA in Western blots. One hour of hypoxia and two hours reoxygenation were not able to change protein expression, neither CD31 nor α-SMA (data not shown). Since three hours are a short time frame to sufficiently change transcriptional and translational processes, we decided to prolong times of hypoxia up to three days. As illustrated in Figure 5, three days hypoxia increased α-SMA expression to 141.7 ± 13.1% (*p* ≤ 0.05 vs. normoxic control, *n* = 8). Inhibition of the TGFβ/SMAD2 pathway by the ALK5 inhibitor SB431542 (1 μM) abrogated EndoMT under hypoxia. Furthermore, stimulation of MVECs with 0.01 ng/mL TGFβ_1_ increased α-SMA expression to 153.1 ± 25.8% (Figure 5), and 1 ng/mL TGFβ_1_ to 252.0 ± 44.7% (Figure 5) (each *p* ≤ 0.05 vs. control, *n* = 8). The expression of CD31 did not change under hypoxia or 0.01 ng/mL TGFβ_1_ (Figure 5). Only stimulation of endothelial cells with high amounts of TGFβ_1_ (1 ng/mL) reduced CD31 expression to 64.4 ± 5.0% (*p* ≤ 0.05 vs. control, *n* = 8) (Figure 5). 

**Figure 6:**
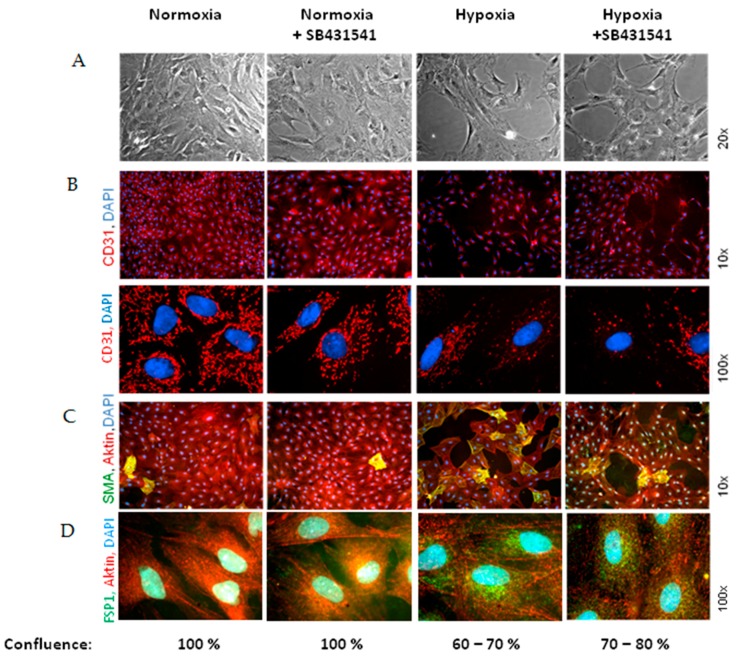
Hypoxia promotes EndoMT via ALK5 receptor. Endothelial cells were exposed to 3 days normoxia or hypoxia in presence or absence of the ALK5 inhibitor SB431542 (1 μM). (**A**) Phase contrast pictures. In these pictures confluence of the cell layer was determined. Additionally, immnuofluorescene was performed: (**B**) double staining with CD31 antibodies (red) and nuclear DAPI-staining (**C**) triplicate staining with α-SMA antibodies (green), actin staining with phalloidin (red), and nuclear DAPI staining (blue), (**D**) triplicate staining with FSP1 antibodies (green), actin staining with phalloidin (red), and nuclear DAPI staining (blue).

Similar changes as in Western blots could be observed in immunohistology (Figure 6). After three days of hypoxia all cells still expressed CD31, although the distribution of CD31 all over the cell surface was reduced after hypoxia (Figure 6B). The number of α-SMA positive cells increased to 19.5 ± 7.4%, compared to 8.9 ± 2.2% in normoxic controls (*p* ≤ 0.05 vs. control, *n* = 5, 800–1000 cells counted). Interestingly, the number of α-SMA-positive cells was reduced by the ALK5 inhibitor SB431542 (1 μM) (Figure 6C), but the localization of CD31 was not influenced by ALK5-inhibition (Figure 6C). As an additional marker for mesenchymal transition we observed increased FSP1 expression in MVECs after 3 days hypoxia (Figure 6D). FSP1 predominantly accumulated close to the nucleus. This FSP1 accumulation was reduced by ALK5 inhibition.

Besides the enhanced α-SMA and FSP1 expression which is characteristic for EndoMT, a disruption of the cell monolayer was observed in phase contrast microscopy under hypoxic conditions (Figure 6A). Thereby confluence of the cell monolayer was reduced by around 30%. Formation of pore-like structures was detected in the monolayer. Especially at these pore forming structures MVECs lost their round to oval cell morphology and turned into elongated, spindle-like structures. Predominantly, α-SMA positive cells were found around the pores. Pore formation was only minimally reduced by the ALK5 inhibitor SB431542 (1 μM). 

**Figure 7:**
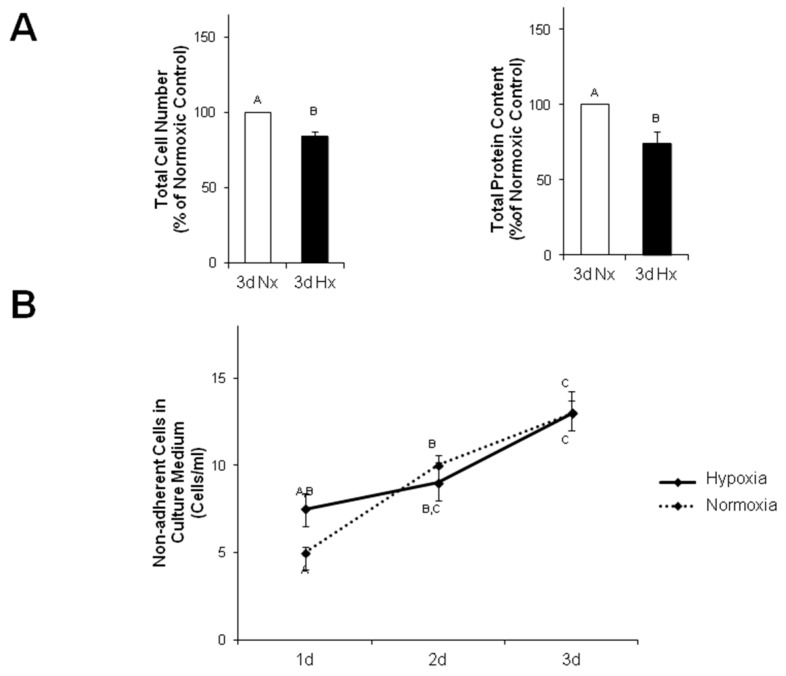
Reduced number of MVEC after 3 days of hypoxia. (**A**) After three days of normoxia or hypoxia total amount of MVECs (*n* = 6) and total protein concentration per culture dish (*n* = 7) were determined. (**B**) After one, two or three days of normoxia or hypoxia, the amount of non-adherent cells per mL culture medium was determined (*n* = 5). Uppercase letters indicate significant differences between groups with *p* ≤ 0.05.

Interestingly, the total cell number per culture dish was reduced to 84.2 ± 2.8% after 3 h of hypoxia (*p* ≤ 0.05 vs. normoxic control, *n* = 6), and the total protein concentration per dish, as determined by Lowry assay, was also reduced to 74.4 ± 7.9% (*p* ≤ 0.05 vs. normoxic control, *n* = 7) (Figure 7A). We speculated that cells may have lost contact/adherence to the culture dish during hypoxia. Therefore, we determined the number of non-attached cells in the culture medium after one, two and three days of hypoxia. Although the number of cells in the culture medium increased during this time from 7.5 ± 0.9 to 13.0 ± 1.2 cell/mL (*p* ≤ 0.05, *n* = 5), this increase was independent of hypoxia, since a similar rise was observed in normoxic controls (Figure 7B).

**Figure 8:**
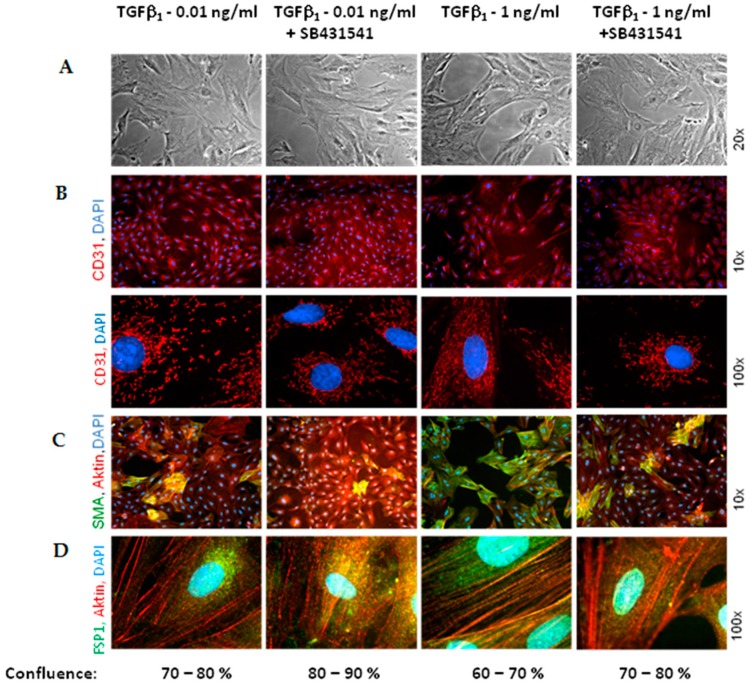
TGFβ_1_ promotes EndoMT via ALK5 receptor. MVECs were exposed to TGFβ_1_ for three days in presence or absence of the ALK5 inhibitor SB431542 (1 μM). (**A**) Phase contrast pictures. In these pictures confluence of the cell layer was determined. Additionally, immnuofluorescene was performed: (**B**) Double staining with CD31 antibodies (red) and nuclear DAPI-staining (**C**) Triplicate staining with α-SMA antibodies (green), actin staining with phalloidin (red), and nuclear DAPI staning (blue), (**D**) triplicate staining with FST1 antibodies (green), actin staining with phalloidin (red), and nuclear DAPI staining (blue).

Addition of TGFβ_1_ resembled the situation of hypoxic endothelial cells in concern to the enhancement of α-SMA- and FSP1-positive cells (Figure 8C,D). While 0.01 ng/mL TGFβ_1_ provoked similar changes as three days of hypoxia, addition of 1 ng/mL TGFβ_1_ dramatically converted endothelial cells into α-SMA-positive cells. Nearly 100% of cells were α-SMA positive after three days exposure to 1 ng/mL TGFβ_1_. Enhancement of α-SMA- and FSP1-expressing cells was reduced by the ALK5 inhibitor. At the same time as α-SMA/FSP1 positive cells increase, pore formation became visible in the cell monolayer, although the pores present more irregular structures as under hypoxia (Figure 8A). ALK5 inhibition prevented pore formation under TGFβ_1_ slightly. Interestingly, changes in CD31 localization were not evident under TGFβ_1_ (Figure 8B).

## 3. Discussion

The main findings of the study are that hypoxic conditions provoke endothelial mesenchymal transition of MVECs via TGFβ_1_/SMAD2 signaling, and induction of pore forming structures in the endothelial cell monolayer. Furthermore, paracrine apoptosis induction in cardiomyocytes could be shown, that is induced via the release of TGFβ_1_ from hypoxic/reoxygenated MVECs. Therefore, oxygen deprivation of MVECs in myocardial infarction may provoke adverse outcomes by contributing to cardiac fibrosis and loss of cardiomyocytes.

Under hypoxic conditions, we could show enhanced expression of TGFβ_1_ precursor protein and activation of SMAD1/5, and 2. Thus, hypoxia induces the classical TGFβ pathway in endothelial cells. SMAD2, but not SMAD1/5 activation could be blocked by an inhibitor of the TGFβ_1_ receptor ALK5, thereby indicating specificity of this inhibitor for TGFβ-SMAD2 signaling. 

As marker for EndoMT, we determined expression of the mesenchymal markers α-SMA and FSP1, which increased under hypoxia and TGFβ_1_ stimulation of MVECs. Since their expression was blocked by the ALK5-inhibitor, we can conclude that EndoMT under hypoxia is mediated via TGFβ_1_/SMAD2 signaling. Interestingly, stimulation of MVECs with a very low concentration of TGFβ_1_ (0.01 ng/mL) resembled the increase in α-SMA- and FSP1-positive cells under hypoxia, whereas higher TGFβ_1_ concentrations (1 ng/mL) provoked a much stronger effect and turned nearly all MVECs into α-SMA positive cells. This indicates that only a minimal release of TGFβ_1_ in the heart can trigger EndoMT. Our findings are in accordance with studies from the group of Zeisberg and coworkers that have demonstrated in human coronary and microvascular endothelial cells induction of EndoMT via TGFβ/SMAD2/3 signaling in a similar time frame of three hours hypoxia [2]. Also here, strong induction of HIF-1α and α-SMA expression and increased numbers of α-SMA-positive cells were shown. 

Interestingly, we did not observe a change in the expression of the endothelial marker CD31, neither under hypoxia nor under low TGFβ_1_ concentrations. All cells expressed CD31, even under high dose of TGFβ_1_. Therefore, the endothelial cell origin after EndoMT can still be detected via CD31 expression. 

Besides the enhancement of α-SMA/FSP1 positive cells, we detected a derangement of the endothelial cell layer in hypoxic MVEC cultures. This effect was not described by Xu et al. [2] under similar hypoxic conditions. Reasons for this discrepancy may lie in the low confluence of the cells that was used in those studies. In addition, the cells used in the study of Xu and coworkers had experienced a much higher passage number in cell culture that may contribute to dedifferentiation. In our study we used freshly isolated MVECs from rat hearts with only one passage step and a confluence of 95%. In addition to the normal isolation protocol for MVECs [12], we now optimized the time of collagenase digestion and cell adhesion time, so that we achieved over 90% purity of MVECs. Under this condition we observed pore forming units in the hypoxic cell layer. This hypoxic pore formation may contribute to increased microvascular permeability after myocardial infarction. Gündüz et al. [3] have shown that ATP metabolites (AMP and adenosine), that accumulate in hypoxic cells, enhance permeability in MVECs due to loss VE-cadherin at cell–cell junctions, leading to cell shrinkage and derangement of the endothelial cell layer. Similar findings are presented by Aslam et al. [13] in hypoxic reoxygenated porcine aortic endothelial cells. We did not investigate VE-cadherin localization in hypoxic MVECs. But CD31, as another adhesion protein did not change in our system. Therefore, CD31 did not play a role in pore formation under hypoxia or TGFβ_1_. Instead, in our study we observed a reduced cell number in hypoxic MVECs cultures. This decreased cell number cannot be explained by increased numbers of non-adherent cells in the culture medium, since the number of floating, non-adherent cells was similar under normoxic and hypoxic conditions. Interestingly, Härtel et al. [14] have demonstrated an anti-apoptotic behavior of MVECs under hypoxic/reogygenated conditions, so that enhanced cell death could not to be expected. Therefore, we assume that decreased proliferation rates caused diminished cell numbers compared to normoxic controls and may contribute to derangements in the cell layers. Furthermore, EndoMT itself may contribute to pore formation as α-SMA positive cells were predominantly found around these pores. Similarly to hypoxia, TGFβ_1_ provokes pore formation. However, since pores are not completely abolished by ALK5 inhibition, besides SMAD2 other pathways must be involved in derangement of MVECs.

Since the primary function of the vascular endothelium is to form a selective barrier and regulate trafficking of macromolecules and blood cells across the vessel wall [15], pore formation in the endothelial cell layer may facilitate release of growth factors into the cardiac tissue and migration of non-cardiac cells into the tissue. Therefore, endothelial derived mesenchymal cells, as they increase under hypoxic conditions, can easily penetrate the cardiac tissue and contribute to cardiac fibrosis. Furthermore, TGFβ that is synthesized in hypoxic endothelial cells is able to easily invade into cardiac tissue and induce apoptotic cardiomyocyte death, as has been shown in this study with conditioned medium from hypoxic/reoxygenated MVECs. Since the proapoptotic effect in cardiomyocytes of conditioned medium from hypoxic MVECs was reduced by ALK5 inhibition, we can conclude that apoptosis was induced by TGFβ_1_ which was released from hypoxic MVECs. Furthermore the signal is mediated via the classical SMAD2 pathway, which has already been described for apoptosis induction by TGFβ_1_ in cardiomyocytes [16,17]. 

In conclusion, using the advantage of analysis of single cell type endothelial cells from cardiac microvasculature of rats under defined hypoxic conditions, we could demonstrate that severe hypoxia provokes EndoMT and reduction in cell numbers, presumably due to reduced proliferation rates. Both effects contribute to disintegration of the cell layer that may facilitate immigration of endothelial derived mesenchymal cells and release of TGFβ_1_ into the cardiac tissue. These processes can contribute to cardiac fibrosis and loss of cardiomyocytes and finally heart failure progression in vivo. As both processes are mediated via TGFβ/SMAD2 signaling, interference with this pathway should be a major aim for prevention of myocardial damage due to infarction.

## 4. Materials and Methods

The investigation conforms to the “Guide for the Care and Use of Laboratory Animals” published by the National Institutes of Health (NIH Publication No. 85–23, Revised 1996) and the principles outlined in the Declaration of Helsinki (*Cardiovasc* *Res* 35: 2–3, 1997). Use of animals for cell isolation was registered at the Justus-Liebig-University (registration-No.: 419-M).

### 4.1. Cell Isolation and Microvascular Endothelial Cell Culture (MVEC)

MVECs from 200 to 250-g Wistar rats were isolated according to Peters et al. [12]. In brief, hearts were perfused with collagenase (400 mg/mL) for 25 min at 37 °C. After removal of aorta and atria, ventricles were cut with a tissue chopper and cardiomyocytes were separated from remaining cardiac cells by centrifugation. The remaining cells were redigested with collagenase for different times (Figure 1A). 45 min of redigestion time resulted in the highest purity of MVEC. Cells were cultured in a fully humidified atmosphere at 37 °C and 5% CO_2_. 95% confluent cultures of primary endothelial cells were trypsinized in phosphate-buffered saline [PBS, composition in mM: 137 NaCl, 2.7 KCl, 1.5 KH_2_PO_4_, and 8.0 Na_2_HPO_4_, at pH 7.4, supplemented with 0.05% (*w*/*v*) trypsin, and 0.02% (*w*/*v*) EDTA] and seeded at a density of 2.6 × 10^3^ cells/cm^2^. Cells were cultured in cell basal medium 199 with Earle’s salt, supplemented with 100 IU/mL penicillin G, 100 μg/mL streptomycin, and 10% (*v*/*v*) NCS and 10% (*v*/*v*) FCS, respectively. Experiments were performed three to four days after seeding, with a confluence of ~95%. Schematic isolation protocol is depicted in Supplementary Figure S1.

### 4.2. Cell Isolation and Ventricular Cardiomyocyte Cultures

Ventricular cardiomyocytes were isolated from 200 to 250 g male Wistar rats, suspended in basal culture medium and plated on culture dishes, which were preincubated overnight with 4% fetal calf serum in medium 199, as previously described [18]. The basal culture medium (CCT) was modified medium 199 including Earle’s salts, 2 mM L-carnitine, 5 mM taurine, 100 IU/mL penicillin, 100 μg/mL streptomycin and 10 μM cytosine-β-d-arabinofuranoside (pH 7.4). Three hours after plating, the dishes were washed twice with CCT medium. This results in cultures of about 90% quiescent rod-shaped cells on average.

### 4.3. Experimental Protocol for Hypoxic Cell Culture

Hypoxic culture conditions were generated by exposure of cells to a constant stream of humified 95% N_2_/5% CO_2_ in a gas-tight chamber (BioSpherix, Lacona, NH, USA) at 37 °C for one hour to 3 days. O_2_ content was continuously monitored by an oxygen sensor (ProOx P110, BioSpherix, Lacona), and was below 1% O_2_. In parallel, normoxic control cells were cultured under the same conditions and times, but with 5% CO_2_. Inhibitors were applied 30 min before hypoxic or normoxic treatment. A scheme of the experimental time protocol for hypoxia/reoxygenation is depicted in Supplementary Figure S2.

### 4.4. Immunoblot Analysis

Proteins were extracted by homogenization of cells in RIPA buffer (50 mmol/L Tris/HCl, pH 7.5, 150 mmol/L NaCl, 1% Nonidet P-40, 0.5% deoxycholat, 0.1% SDS, 1 mM PMSF, 1 mM EDTA, 1 mg/L pepstatin). Nucleic acids were digested with benzonase (1% (*v*/*v*)). Samples were denatured in Laemmli buffer at 90 °C for five minutes, loaded on 12.5% SDS-gels, and blotted on PVDF membranes. The following primary antibodies were used: anti-CD31, anti-HIF-1α, anti-α-SMA (Abcam, Cambridge, UK), anti-P-SMAD1/5 and anti-P-SMAD2 (Cell Signaling), anti-von Willbrandt factor (Santa Cruz), TGFβ_1_ (Cell Signaling), and anti-vinculin (Sigma, Taufkirchen, Germany). Protein bands were detected by horseradish peroxidase-labeled secondary antibodies (anti-mouse HRP-linked from Santa Cruz and anti-rabbit HRP-linked from Abcam) using SuperSignal^®^ West Pico-Chemiluminescent Substrat (Thermo Scientific, Darmstadt, Germany) as detection system. Specific signals were normalized against vinculin.

### 4.5. Immunohistochemistry

MVECs, grown on glass coverslips, were treated with methanol at −20 °C for 15 min, or with 4% (*w*/*v*) paraformaldehyde in PBS for 20 min at room temperature for fixation, and washed afterwards with PBS. Fixed cell layers were blocked with PBS containing 10% (*w*/*v*) BSA, primary antibodies were incubated for two hours at room temperature or overnight at 4 °C. After washing was completed, Cyt2- or rhodamin-labeled secondary antibody (Biomol) was applied for one hour. Rhodamine-conjugated phalloidin (300 units/900 μL methanol stock solution in PBS (5/95 (*v/v*)) was used for staining of actin cytoskeleton. 4′,6-Diamidino-2-phenylindol-dihydrochlorid (DAPI, 100 ng/mL PBS) was used for nuclear staining. After washing was completed, coverslips were mounted in glycerol-PBS (90:10, *v*/*v*) and analyzed by confocal microscopy (Zeiss, LSM 510 Meta).

### 4.6. Detection of Chromatin Condensation

Twenty four hours after apoptosis induction, cardiomyocytes were stained for 30 min with Hoechst 33258 (5 mg/L) and propidium iodide (1 mg/L). Hoechst 33258 is a membrane-permeable DNA dye that stains apoptotic, condensed nuclei more intensively. Propidium iodide only stains nuclei of necrotic cells, since it is unable to pass the cell membrane of intact or apoptotic cells. Cells were analyzed by fluorescence microscopy. For quantification of apoptosis and necrosis, 200 randomly distributed cells were counted in each experiment. 

### 4.7. Statistics

Data are given as means ± standard errors (S.E.) from *n* different culture preparations. Statistical comparisons were performed by Student–Newman–Keuls test for post hoc analysis (Godfrey, 1985) or student’s *t*-test, if only two parameters were compared. A *p*-value of less than 0.05 was considered statistically significant. SPSS software was used for statistical analysis.

## Supplementary Materials

Supplementary materials can be found at www.mdpi.com/1422-0067/18/11/2290/s1.
