# Supplementary material for: Endothelial Mesenchymal Transition in Hypoxic Microvascular Endothelial Cells and Paracrine Induction of Cardiomyocyte Apoptosis Are Mediated via TGFβ1/SMAD Signaling
Source: Int J Mol Sci. 2017 Oct 31;18(11):2290. doi: 10.3390/ijms18112290 (PMC5713260; doi:10.3390/ijms18112290)
Supplement: Supplementary file 1 [file ijms-18-02290-s001.pdf]

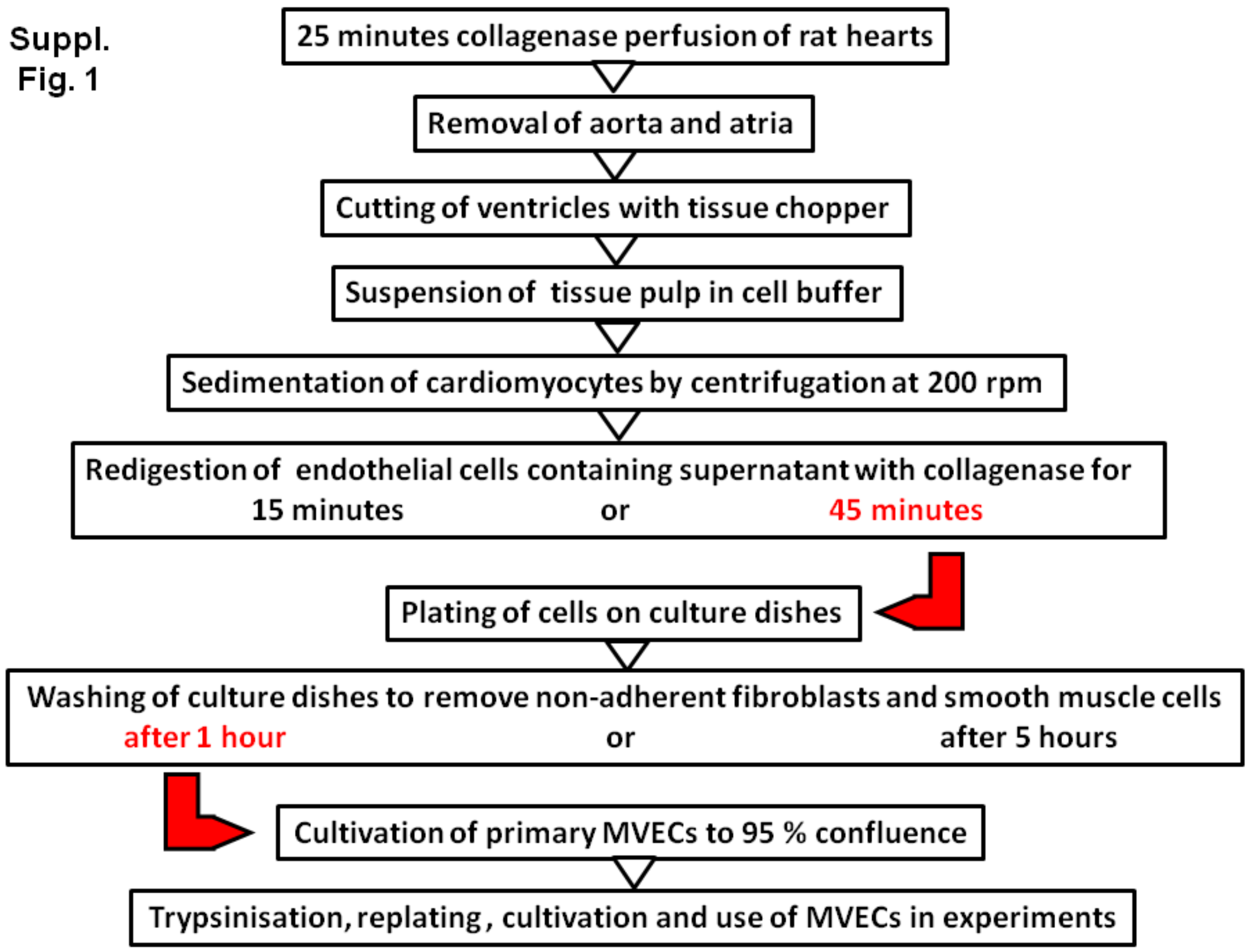

Suppl. Fig. 1: Isolation protocol for pure microvascular endothelial cells (MVECs)
Using different times of collagenase redigestion and adherence of cells to culture dishes, purity of MVEC cultures could be optimized. Optimal duration for redigestion for 45 minutes, and washing of cultures 1 hour after plating are indicated in red.

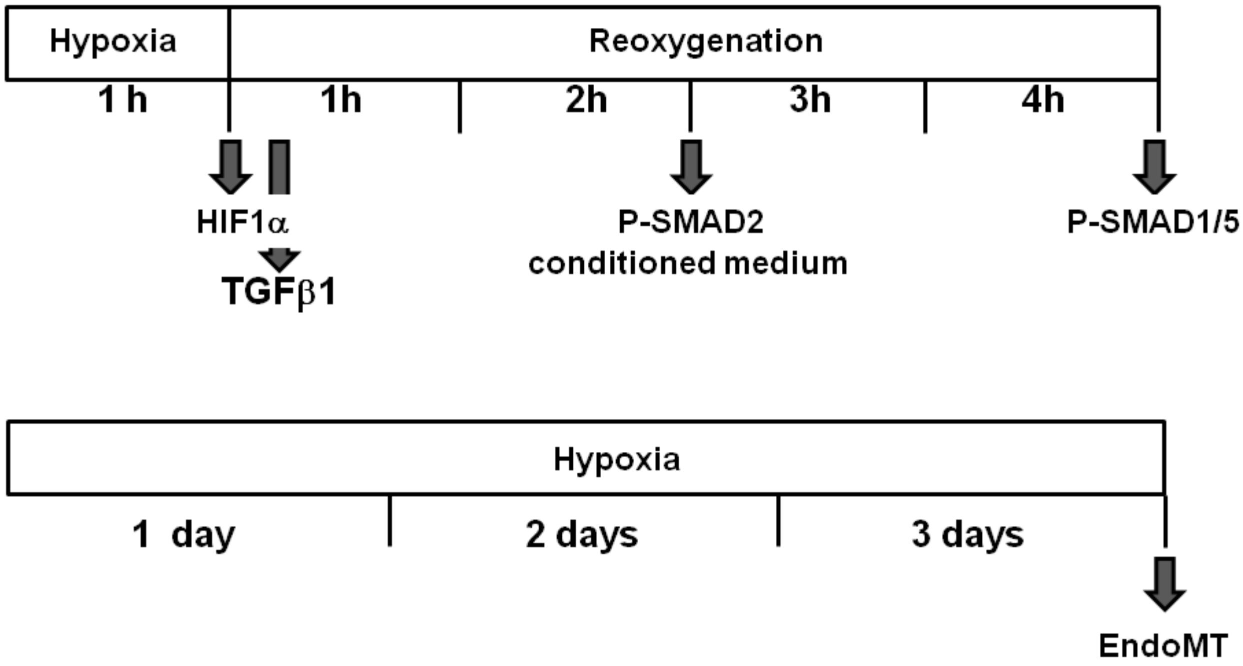

Suppl. Fig. 2

Suppl. Fig. 2: Experimental design of the study

Since signaling events are induced in a sequential order, MVECs were exposed to different times of hypoxia and reoxygenation in order to detect induction/activation of diverse signaling molecules and processes. HIF-1α is detected after 1 hour of hypoxia, TGFβ_1 after 1 hour hypoxia and 15 minutes of reoxygenation. SMAD2 phosphorylation is evident after 1 hour hypoxia and 2 hours of reoxygenation, and SMAD1/5 phosphorylation after 1 h hypoxia and 4 hours of reoxygenation. Conditioned medium from MVECs were collected after 1 hour hypoxia and 2 hours of reoxygenation. EndoMT was analyzed after 3 days of hypoxia.
